# Lung cancer incidences after liver transplantation: A systematic review and meta‐analysis

**DOI:** 10.1002/cam4.6265

**Published:** 2023-06-23

**Authors:** Chang Fu, Xiaocong Li, Yongjin Chen, Xiaoyin Long, Kai Liu

**Affiliations:** ^1^ Department of Hepatobiliary and Pancreatic Surgery, General Surgery Center First Hospital of Jilin University Changchun China; ^2^ Medical Research and Biometrics Center, National Clinical Research Center for Cardiovascular Diseases, State Key Laboratory of Cardiovascular Disease, Fuwai Hospital, National Center for Cardiovascular Diseases, Chinese Academy of Medical Sciences and Peking Union Medical College Beijing China

**Keywords:** liver transplantation, lung cancer, meta‐analysis, standardized incidence ratio

## Abstract

**Background:**

Liver transplantation has made significant progress in recent decades. Lung cancer is one of the most frequently occurring cancers after liver transplantation. However, the risk of lung cancer among liver transplant patients compared with the general population is unclear. The aim of this meta‐analysis was to assess the risk of developing lung cancer after liver transplantation.

**Methods:**

All eligible studies published in PubMed, Web of Science, and Embase from database inception to April 2022 were included. Standardized incidence ratio was used to describe the increased risk of lung cancer in liver transplant recipients as compared with the general population. The random‐effects model was used for the calculations. A funnel plot and Egger test were performed to assess the potential publication bias.

**Results:**

Our meta‐analysis included 15 studies, which involved 76,897 liver transplantation patients. Studies included in this review showed significant heterogeneity (*I* ^2^ = 65.3%; *p* < 0.001), which required a random‐effects model for effect pooling. The results indicated a significant higher risk of developing lung cancer in liver transplant patients than the general population with a pooled SIR of 2.06 (95% CI: 1.73, 2.46, *p* < 0.001). When stratified by region, no significant regional difference was observed. It showed a similarly doubled risk of lung cancer in Europe and North America, but an insignificantly increased risk in Asian populations. The sensitivity analysis by removal and substitution of each literature did not change the results.

**Conclusion:**

Our meta‐analysis suggests that liver transplant patients are twice as likely as the general population to develop lung cancer. Further research on risk factors for the development of lung cancer after liver transplantation should be conducted and appropriate surveillance protocols should be developed to reduce the risk of its occurrence.

## 1 INTRODUCTION

Liver transplantation (LT) has proven to be an effective treatment modality for patients with acute liver failure, end‐stage liver disease, or liver cancer in past decades. In 2020, a total of 8906 liver transplants were performed in the United States.^1^ With the advancement in surgical techniques, improvement of postoperative strategies, and optimization of immunosuppressive therapies, the survival outcome for liver transplant patients has been significantly improved. In Europe, the 1‐ and 5‐year overall survival rates of LT patients have reached 86% and 74%, respectively.^2^ The prolonged survival of LT patients has gradually shifted people's attention to the long‐term prognosis of patients.

The development of post‐transplant malignancies is the main cause of death after LT, and the increased incidence is thought to be a consequence of the immunosuppressive therapy after transplantation which may affect tumor development and progression through a variety of mechanisms.^3^, ^4^, ^5^, ^6^ It has been reported that lung cancer is one of the most common solid organ tumors after LT.^7^ Engles et al. showed that lung cancer risk after LT was nearly 2‐fold compared with the general population.^8^ A Swiss study also suggested a marked increase in lung cancer risk after LT.^9^ However, other studies did not find significant differences in the risk of lung cancer between LT patients and the general population.^10^, ^11^, ^12^, ^13^ The degree of increased risk of lung cancer in liver transplant patients compared with the general population is unclear. Differences in study designs, sample sizes, follow‐up lengths, and immunosuppressive medication might account for the inconsistent outcomes among these studies. Thus, the definite association between lung cancer and LT has not been fully established. Whether and to what extent the risk of lung cancer is increased in LT recipients as compared with the general population is still uncertain.

By conducting this systematic review and meta‐analysis, we aimed to determine the change in the lung cancer incidences after LT compared with the general population.

## 2 METHODS

### 2.1 Materials and methods

This systematic review was performed in accordance with the PRISMA (Preferred Reporting Items for Systematic Reviews and Meta‐Analysis) (Table S1) and AMSTAR (Assessing the methodological quality of systematic reviews) Guidelines.^14^ This research is registered on PROSPERO (CRD42022326101) with methods established prior to conducting the review.

### 2.2 Data sources and search strategy

The authors conducted an independent systematic literature search in PubMed, Embase, and Web of Science databases from inception to April 2022. Search keywords included: “liver” OR “hepatic” AND “transplant” OR “transplantation” AND “lung cancer” OR “pulmonary cancer”. The titles and abstracts were independently reviewed to eliminate duplicate reports and unrelated literature. Then, the full text was read to identify the eligible studies using the inclusion and exclusion criteria outlined below. And the references of all included studies and relevant review articles were also screened for eligible studies.

### 2.3 Eligibility criteria

Study inclusion criteria were as follows: (i) be an observational cohort study where researchers observe participants and track health outcomes of interest (the occurrence of lung cancer for this study) over time; (ii) be published in English; (iii) the research population are patients undergoing LT; (iv) report the standardized incidence ratios (SIR) of lung cancer with 95% confidence intervals (CI) in liver transplant population compared with the general population. The following exclusion criteria were used to exclude articles from the meta‐analysis: (i) written in languages other than English; (ii) involved animal experiments; (iii) case reports, literature reviews, or conference abstracts; (iv) including patients with failed LT; and (v) had incomplete data. In addition, we found more than one article based on the same database but covering different regions or recruiting periods. To account for this situation, the studies with the largest coverage or sample size were included in the main analysis.

All studies were independently reviewed by two authors. Disagreements were resolved with the help of a third researcher.

### 2.4 Data extraction and quality assessment

The quality of the included studies was evaluated separately by two independent researchers based on the Newcastle‐Ottawa Scale (NOS). The NOS scale, with a full score of 9, consists of 8 items in 3 dimensions: 4 items for subject selection, 1 item for comparability and 3 items for outcome. A maximum of 1 point is awarded to each item, except for comparability, which is assigned a maximum of 2 points. A higher score indicates higher research quality and studies with a score of ≥6 are considered eligible for the analysis. The relevant information to be extracted includes: the first author, publication year, nature of the study, country of study, sample size, age, gender, and follow‐up time, lung cancer cases after LT, SIR with 95% CIs reported in LT recipients, etc. Any disputes in the process of information extraction and quality assessment will be consulted and confirmed with the third researcher.

### 2.5 Statistical analysis

SIR was chosen to measure the relative risks of lung cancer in patients compared with the general population. The SIR is defined as the ratio of the observed and expected number of lung cancer and the expected number of incident cancers is calculated by applying the cancer incidence rate (person‐years) of the general population in LT patients. A SIR of >1 suggests increased risk, and a SIR of <1 indicates decreased risk. We log‐transformed the SIR and its 95% CI for each study to calculate the pooled transformed estimates based on the normal distribution. Then, an inverse transformation was applied to the pooled transformed SIR to obtain a pooled estimate on the original scale.

Statistical heterogeneity among the included studies was assessed using Cochran's Q statistic and Higgins' *I* ^2^ statistic. A random‐effects model was preferred if the heterogeneity was large (*p* < 0.10 or *I* ^2^ > 50%), while a fixed‐effects model was used if the heterogeneity was small (*I* ^2^ ≤ 50% and *p* ≥ 0.10). A funnel plot and Egger test were used to assess the potential publication bias. Subgroup analysis was performed on the occurrence of lung cancer after LT according to study region, follow‐up time, presence of liver cancer before LT, and whether to report the use of immunosuppressive agents. Low‐quality studies (NOS score <6) were excluded from sensitivity analyses. Furthermore, we removed one study each time and recalculated the pooled risk estimates to assess the robustness of our results. Stata 16.0 (Stata, College Station, Texas, USA) was used for all analyses.

## 3 RESULTS

### 3.1 Study selection

**FIGURE 1:**
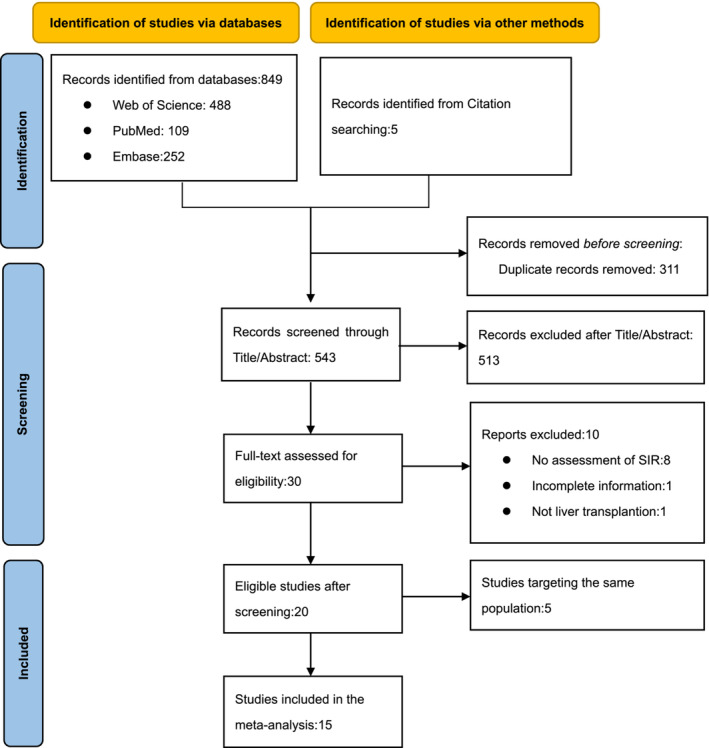
The flow diagram shows the selection process according to the PRISMA statement.

A total of 849 studies were retrieved from the databases (including 488 from Web of science, 109 from PubMed, and 252 from Embase) while five articles were obtained from other sources. After deduplicating and reviewing title or abstract, 30 studies were retained for further full‐text screening. And 20 literatures met the inclusion and exclusion criteria. One study in Taiwan, three in Italy, and one in Finland were excluded because they used the same database but had a small sample size. Finally, 15 articles were included in our meta‐analysis (Figure 1).

### 3.2 Study characteristics

The 15 eligible studies involved 76,897 patients and had been conducted between 1970 and 2014. Of the 15 studies included in the final analysis, 11(73.3%) of them were conducted in Europe, two (13.3%) in North America, and two (13.3%) in Asia. 13 (86.7%) studies were prospective, and 2 (13.3%) were retrospective. The LT and cancer data of eight studies were obtained from national or regional registries with good representation. Three studies used data from multiple medical centers, and four studies used data from a single center, which resulted in a relatively poor representation. The lowest SIR was reported from Taiwan (SIR = 0.9) and the highest was from Swiss.Other studies reported SIR ranging from 1.4 to 6. Mean age and follow‐up time were not reported in some studies, as shown in Table 1. Seven studies reported data on the uses of immunosuppressive agents, with combinations of calcineurin inhibitors (CNI) and antimetabolites or mammalian target of rapamycin inhibitors (mTOR) being the most common ones. The results of the quality assessment showed that the NOS scores of these studies were higher than or equal to 6 points, which indicated high quality and could be included in the analysis (Table S2).

**TABLE 1:** Characteristics of included studies.

Study	Nature	Region	Transplantation type	Time period	Data source	Age (years)	Male (N/%)	No. of LT	Incident cases	Follow‐up time	Matching variables in SIR calculation	SIR (95%CI)	Journal
Yeh, 2020^39^	Prospective	Taiwan	Heart, kidney, and liver	1997–2011	National Health Insurance Research Database	Mean ± SD: 45.5 ± 18.0	1523/71.6	2127	5	Mean ± SD: 4.2 ± 2.7 years	Age, sex, and year	0.9 (0.4, 2.3)	Exp Clin Transplant
Emanuel, 2019^9^	Prospective	Swiss	SOT	2008–2014	Swiss Transplant Cohort Study	Median (IQR): 54.4 (43.9, 61.2)	365/65.5	557	5	Median (IQR): 2.7 (1.2, 4.5) years	——	6 (1.93, 14)	Swiss Med Wkly
O'Neill, 2019^12^	Prospective	Ireland	SOT	1994–2014	St Vincent's University Hospital +the National Cancer Registry Ireland	Mean (range): 48.5 (15.7–70.7)	304/54.1	562	5	Not reported	Age, sex, and year	1.51 (0.49, 3.53)	Clin Transplant
Taborelli, 2018^32^	Retrospective	Italy	Liver	1985–2014	Nine centers from all over Italy	Median (IQR): 53 (46,59)	2113/74.6	2832	28	Median (IQR): 5.4 (2.4, 10.0) years	5‐year age group, sex, area of residence, and calendar period	1.4 (1.0, 2.1)	Int J Cancer
Nordin, 2018^19^	Prospective	Nordic countries	Liver	1982–2013	the Nordic LT Registry+ national cancer registries	Mean ± SD: 49 ± 12	2390/56	4246	30	Median: 6.6 years	5‐ year age groups, calendar year, and country	1.89 (1.28, 2.69)	Am J Transplant
Seree, 2018^17^	Prospective	France	Liver	1993–2012	the French Agence de la Biomédecine+ Cristal	Median (IQR): 50.1 (40.3, 57.4)	7325/65.3	11,226	188	Mean ± SD: 6.1 ± 4.3 years	Age in 5‐year increments, sex, and calendar year	2.56 (2.21, 2.95)	Liver Tranpl
Heo, 2017^15^	Prospective	South Korea	Liver	2010–2014	HIRA database	Median: 53	2017/81.9	2462	17	Median (range): 12.4 (0.8–53.0) months	——	4.94 (2.88, 7.91)	Hepatol Int
Krynitz, 2013^11^	Prospective	Sweden	Kidney, liver, heart, and lung	1970–2008	Swedish National Patient Register +Swedish Cancer Register	Median (IQR): 49 (36, 57)	695/57	1221	6	Median (range): 5 (0–21) years	Age, sex, and calendar period	1.8 (0.7, 4.0)	Int J Cancer
Scherem, 2013^40^	Retrospective	Germany	Liver	1983–2010	Hannover Medical School	Median (range): 42(0–71)	1060/53.3	2000	14	Median: 7.25 years	Sex and age	1.85 (1.11, 3.10)	Liver Tranpl
Engels, 2011^8^	Prospective	USA	SOT	1987–2008	SRTR with 13 US population‐based cancer registries	Median: 47	23,112/60.9	37,888	300	Not reported	Sex and age	1.95 (1.74, 2.19)	JAMA
Herrero JI, 2011^34^	Prospective	Spain	Liver	1990–2009	——	Without SRM: 55.56 ± 9.87 With SRM: 60.06 ± 6.60	Without SRM: 76.0% With SRM: 96.2%	339	9	Mean:7.5 years	Sex and age	2.17 (0.99, 4.12)	Liver Transpl
Collett, 2010^41^	Prospective	UK	Kidney, liver, heart, and lung	1980–2007	UK Transplant Registry +9 cancer registrations	57% of recipients were 35–59	3592/52.5	6846	Not reported	Median (range): 16 (8–26) years	Five year age groups, gender, and year	1.6 (1.2, 2.2)	Am J Transplant
Finkenstedt, 2009^18^	Prospective	Austria	Liver	1982–2007	Innsbruck Medical University	Mean (range): 53.0 (15.1–76.4)	544/69.8	779	17	Median (range): 4.1 (0–24) years	Age, sex and calendar year	3.1 (1.8, 5.0)	Am J Transplant
Jiang, 2008^10^	Prospective	Canada	Liver	1983–1998	CORR database	62% of recipients were 40–70 years	1238/53.1	2034	10	Mean ± SD: 42.2 ± 33.8 months	Age, sex, and calendar period	1.4 (0.7, 2.6)	Liver Transpl
Oo, 2005^42^	Prospective	England and Wales	Liver	1982–2004	Queen Elizabeth Hospital	60% of recipients were 45–65 years	849/47.8	1778	14	Median (range): 65.6 (32.5–76.9) months	Age, sex, and calendar year	1.96 (1.07, 3.29)	Transplantation

Abbreviations: CI, confidence interval; SIR, standardized incidence ratios; SOT, Solid organ transplant.

### 3.3 Pooled estimates

**FIGURE 2:**
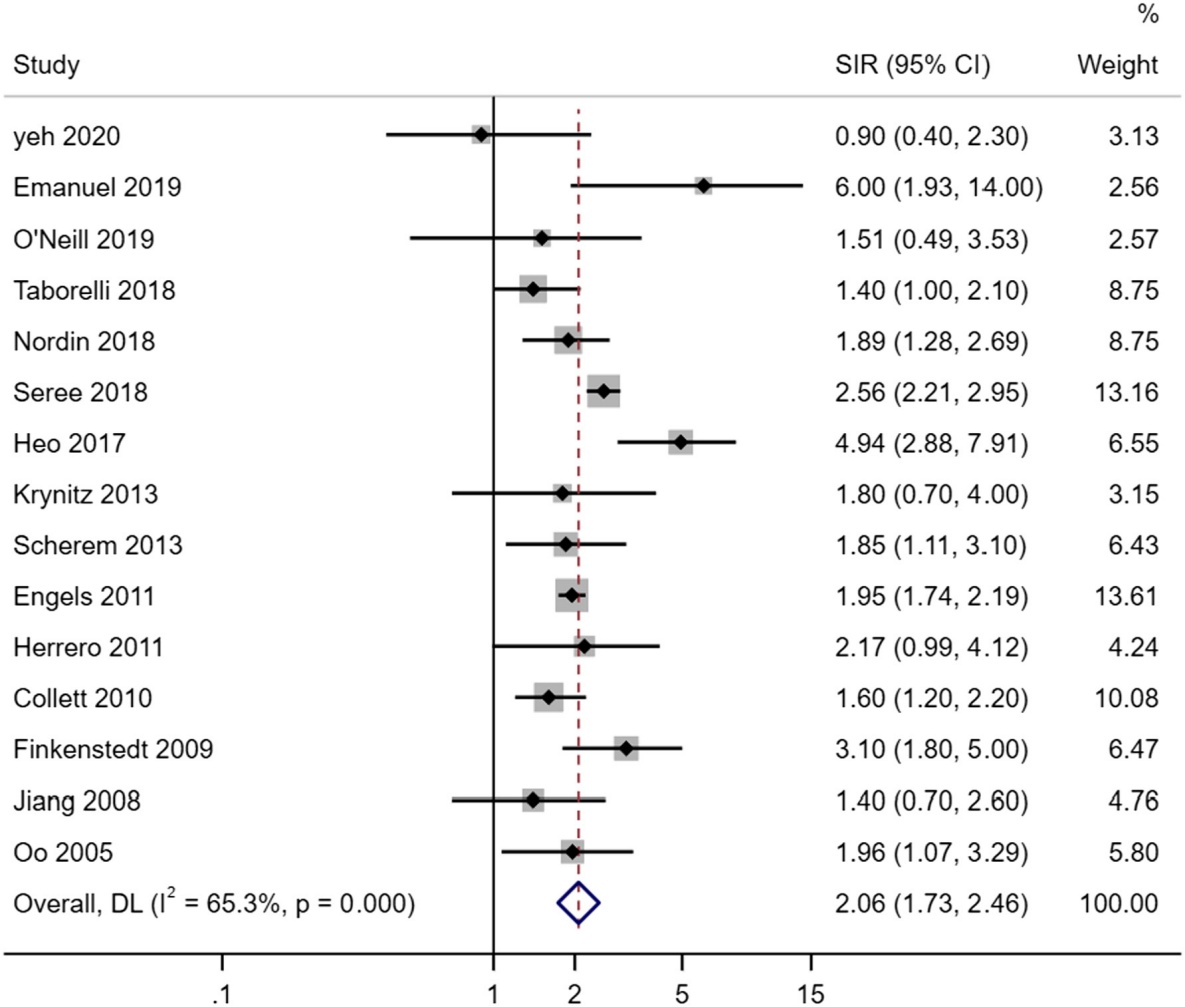
The forest plot of the pooled standardized incidence ratios (SIR) and 95% confidence intervals (95% CI) for lung cancer after liver transplantation. NOTE: Weights are from random‐effects model.

**FIGURE 3:**
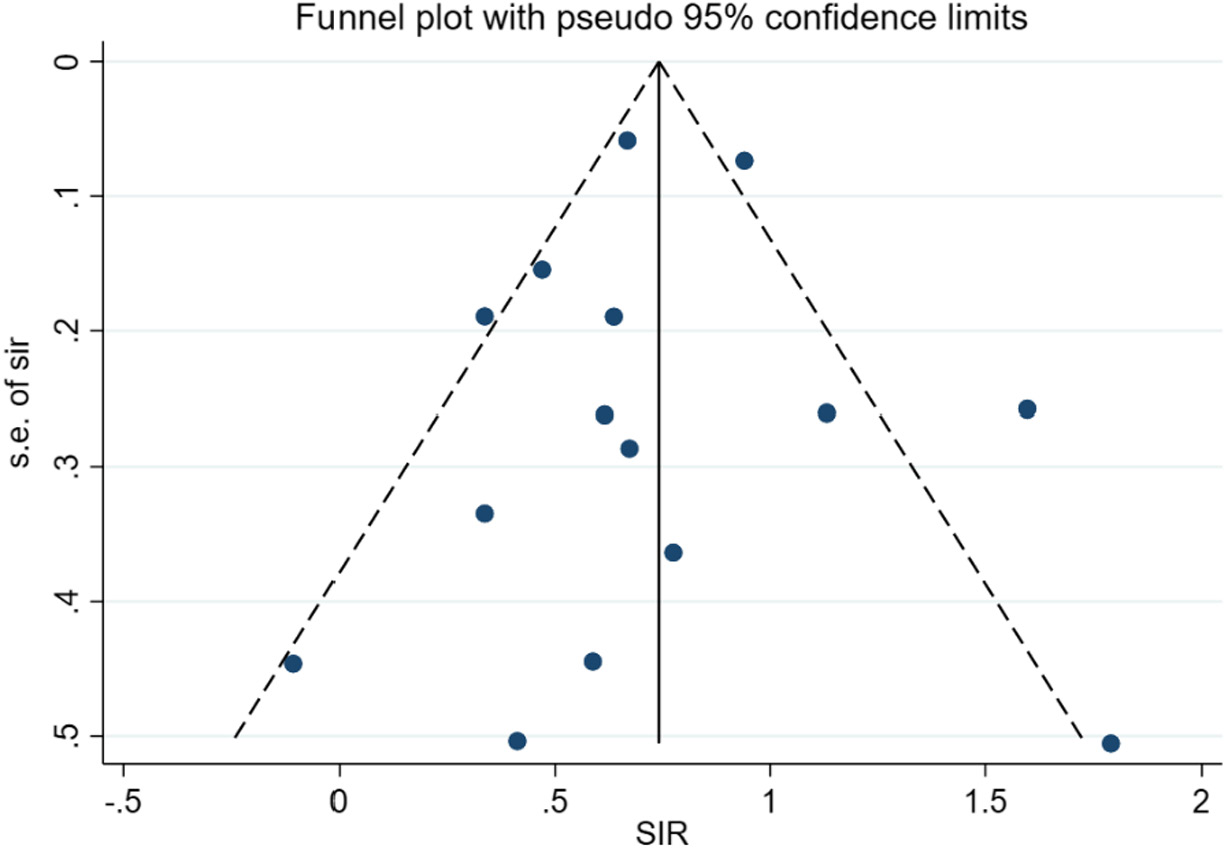
Funnel plot for evaluating publication bias.

These studies exhibited moderate heterogeneity with an *I* ^2^ of 65.3% (*p* < 0.001), therefore, a random‐effects model was used for analyses. In the random‐effects meta‐analysis of the 15 independent studies, the SIR of lung cancer in liver transplant patients was 2.06 (95% CI: 1.73, 2.46, *p* < 0.001) (Figure 2). Funnel graph and Egger test did not reveal any evidence of publication bias (*p* = 0.670) (Figure 3).

### 3.4 Subgroup analysis

Subgroup analyses were performed for regions, follow‐up time. Whether there is cancer before transplantation and the report of immunosuppressive agents (Table 2). The *p* value between different regions was 0.876 (*p* > 0.05), indicating that there was no significant difference in the risk of lung cancer in patients after LT from different regions. The results showed an insignificantly increased risk of lung cancer after LT in the Asian population compared with the general population (SIR = 2.19, 95% CI: 0.41, 11.61), while the risk of lung cancer in the European and North American cohort was doubled compared with the general population. The risk of lung cancer increased after LT regardless of follow‐up time (1.91 [1.68, 2.63] for follow‐up time ≥5 years and 2.61 [1.39, 4.92] for <5 years). The exclusion of pretransplant cancer also did not change the increased risk of lung cancer after LT. There was no heterogeneity in the risk of post‐transplant lung cancer between patients with pretransplant cancer and those without pretransplant cancer (P for homogeneity = 0.234). The use of immunosuppressive agents did not affect the risk of lung cancer after transplantation (P for homogeneity = 0.175).

**TABLE 2:** Subgroup analysis between different regions, follow‐up time and subject selection.

Subgroup	Pool SIR (95% CI)	Homogeneity		
		*I* ^2^(%)	*P*‐within	*P*‐between
Region				0.876
Asian	2.19 (0.41, 11.61)	90.8	0.001	
Europe	2.05 (1.67–2.50)	54.2	0.016	
North America	1.93 (1.72, 2.16)	0.0	0.330	
Follow‐up time				0.352
≥5 years	1.91 (1.68, 2.63)	54.7	0.031	
<5 years	2.61 (1.39, 4.92)	77.8	0.001	
Cancer before transplantation				0.234
Yes	2.38 (1.69, 3.37)	57.2	0.039	
No	1.88 (1.56, 2.26)	48.6	0.049	
Immunosuppressive agents report				0.175
Yes	2.53 (1.69, 3.77)	68.3%	0.004	
No	1.86 (1.55, 2.24)	61.9%	0.010	

### 3.5 Sensitivity analysis

The removal of one study each time did not change the results (Table S3). However, the removal of studies by Heo et al.^15^ did explain some of the inter‐study heterogeneity (*I* ^2^ = 55.2%). To assess the stability of results, the other five studies excluded in the last step were included in the sensitivity analysis, and the effects were recombined, respectively. It showed that the substitution of the literatures did not alter the results. (Table S4).

## 4 DISCUSSION

Recent decades have seen significant advances in LT as a curative treatment option for patients with end‐stage liver disease. The first LT was performed by Starzl in 1963.^16^ Since the number of LT patients increased and survival rate after LT improved, studies should focus on long‐term outcomes after LT. The risk for lung cancer development in LT recipients has been evaluated by many studies, including multicenter or single‐institution studies. This meta‐analysis showed that LT patients had a higher risk for developing lung cancer than the general population, with a pooled SIR of 2.06 (95% CI: 1.73, 2.46).

Globally, lung cancer is the most lethal tumor type. In recent decades, lung cancer incidences after LT have been increasing. In a cohort study involving 37,888 LT patients from the United States, it reported a lung cancer incidence of 178.7 per 100,000 person‐years, with a SIR of 1.95 (95% CI: 1.74, 2.19).^8^ Based on their study, lung cancer risks increased over time.^8^ The limitations of this study were that participants included were limited to four major racial/ethnic groups and follow‐up time were not reported. Another national study in France reported 188 cases of lung cancer in 11,226 patients after LT from 1993 to 2012 with a SIR of 2.56 (95% CI: 2.21, 2.95).^17^ In other studies, lung cancer SIR after LT also increased.^18^, ^19^ In addition, we noticed that one study from Taiwan found a SIR for lung cancer of 0.9 with wide CI including 1. This study excluded patients who developed cancer within the first year of LT, unlike other studies, which may lead to a decrease in the number of lung cancer cases observed in liver transplant patients and therefore result in a lower SIR. Fifteen studies involving 76,897 LT patients were included in our study. The SIR with 95% CI for each study was pooled to estimate the results. As compared to the general population, our meta‐analysis suggests a doubled risk of lung cancer after LT. Both subgroup analysis and sensitive analysis did not substantially affect the results, except that insignificantly increased risk was observed in the Asian population. The insignificant results might be due to the small number of subjects analyzed from Asia (including only 4589 people from two studies). Moreover, the *p* value for heterogeneity among the subgroups of regions was 0.876 (*p* > 0.05), indicating that there was no significant difference in the risk of lung cancer in patients after LT from different regions.

Several factors may lead to increased lung cancer risks after LT, with long‐term immunosuppressive treatment being the main risk factor. Immunosuppressive agents are used to maintain graft health, which may induce the loss of immunovigilance. The effects of immune suppression on the occurrence of certain tumor types were first reported in the early 1970s among people treated with antirejection drugs after organ transplantation.^20^ After the 1980s, CNI were widely administered after LT. CNI are still the main maintenance immunosuppressants after LT. CNI such as tacrolimus and cyclosporine upregulate vascular endothelial growth factor and transform growth factor beta 1, which facilitates tumor growth and spread.^21^ Azathioprine is used after LT as an antimetabolite to interfere with nucleotide synthesis. In a single‐institution study involving 772 liver transplant recipients, it found that azathioprine was an independent predictor of tumor development after LT (relative risk: 3.8; 95% CI: 1.7, 8.6).^22^ As a new class of immunosuppressive medication, mTOR has potential antitumorigenic effects by blocking the pathway that is vital for cancer progression.^23^, ^24^, ^25^ Studies have shown that mTOR reduces the occurrence of de novo malignancies after kidney transplantation.^26^, ^27^ And the combination therapy with mTOR can reduce the dose of CNI, which could reduce cumulative immunosuppression. A meta‐analysis indicated that mTOR improves recurrence‐free survival and reduces the recurrence rate compared with CNI in liver transplant patients with hepatocellular carcinoma.^28^ As for LT, the mechanism of mTOR needs more studies to verify. Maximum immunosuppression is required for early transplant, considering the high risk of rejection.^29^ As the time goes by after transplantation, the immunosuppression burden can be reduced. Carenco suggested a dose–response relationship between CNI and the risk of malignancy after LT.^30^ Therapeutic drug monitoring is used to adjust the dose of immunosuppressive agents, which is based on whole blood concentrations.^29^ The application of therapeutic drug monitoring can balance the relationship between rejection and immunosuppression, which is beneficial to the long‐term survival of patients after LT. In addition, a newly developed tool called “cumulative exposure to tacrolimus (CET)”, which is calculated by the area under the trough concentration curve, can also help clinicians formulate immunosuppressive modulation strategies to prevent cancer after LT.^31^ Due to the limited information, in the current studies, it is unlikely to examine the difference of SIR stratified by different immunosuppressive agents. Another important risk factor for de novo malignancies after LT is alcohol‐related liver disease. Alcoholic cirrhosis is a common indication for LT. Carcinogenic alcohol metabolites decrease retinoic acid levels, which have anticarcinogenic effects and suppress the methylation of certain oncogenes, resulting in their increased expressions.^21^ It is also associated with a higher risk of lung cancer development in patients receiving liver transplants who smoke tobacco. Most of the studies included in our meta‐analysis did not report data on smoking history due to large‐scale database settings. The study conducted in Italy reported 29.7% of liver transplant patients had a history of smoking.^32^ According to European guidelines, smoking cessation should be mandatory in all transplant candidates.^33^ In a study of 342 liver transplant patients, the cumulative risk of smoking‐related malignancies was significantly higher than that in the general population.^34^ In spite of the association between smoking and lung cancer, the lack of smoking data had only a limited impact on our aim to quantify lung cancer risk among LT recipients.

In addition, treatment options for lung cancer after LT are limited. Immune checkpoint inhibitors (ICIs) can be used to treat lung cancer, but may be associated with high risks of transplant rejection in patients after LT.^35^, ^36^ It is challenging to balance the ICIs treatment and immunosuppressive therapy in liver transplant recipients.

At present, there are no standardized early detection and surveillance protocols for patients after LT. Early detection of de novo malignancies is essential for liver transplant patients. For lung cancer, low‐dose computed tomography scanning (LDCT) allows for detection at early stages and reduces mortality in high‐risk populations.^37^ Finkenstedt and Herrero also recommended CT screening programs for patients with a high risk of de novo malignancies after LT, such as old patients and smokers. Compared to other lung cancer screening studies in smoker nontransplant recipients, Herrero found the rate of diagnosis of lung cancer in LT patients is higher than 10%.^38^ Our meta‐analysis concluded that the risk of lung cancer after LT is about twice that of the general population. For those who still smoke after LT, the cumulative risk of lung cancer could be higher. As a result, we believe that LDCT for smokers after LT will have significant health economic value. With regard to immunosuppressive medication, long‐term therapies should be customized and adjusted individually. The UK clinical guideline for adult LT strongly recommended that CNI and mTOR doses should be informed by the use of therapeutic drug monitoring.^29^ To minimize cumulative immunosuppression, the use of mTOR and therapeutic drug monitoring could be applied in the future.

This meta‐analysis has some limitations. First, we failed to analyze the effect of the type of immunosuppressive drugs on the risk of lung cancer since the limited data regarding the types of immunosuppressive drugs reported in current articles. Future studies should focus on the effects of different types of immunosuppressive agents on the incidence of lung cancer after LT to provide evidence for the formulation of clinical immunosuppressive regimens. Second, the indication of LT varies from study to study and the type of liver disease suffered prior to LT also has an impact on the risk of lung cancer after LT. Subgroup analysis on liver diseases could not be performed because most studies did not explicitly report liver diseases leading to LT. Considering that patients undergoing LT for liver cancer may be at increased risk of developing lung cancer after surgery, we conducted a subgroup analysis based on whether the study population included patients with liver cancer. Third, the included data from registries did not include personal information, such as smoking history and alcohol consumption. These factors may act as confounding factors to affect the risk of lung cancer in liver transplant recipients and their effects cannot be completely ruled out.

In conclusion, our findings show that liver transplant patients have a 2‐fold likelihood of lung cancer development, compared to the general population. Reliable surveillance protocols are recommended for liver transplant patients. Further studies are needed to explore the risk factors related to lung cancer development, which helps reduce the risk of lung cancer after LT.

## AUTHOR CONTRIBUTIONS

**Chang Fu:** Conceptualization (equal); writing – original draft (lead); writing – review and editing (equal). **Xiaocong Li:** Data curation (lead); methodology (equal); writing – review and editing (equal). **Yongjin Chen:** Data curation (equal); supervision (equal). **Xiaoyin Long:** Data curation (supporting); writing – review and editing (equal). **Kai Liu:** Conceptualization (equal); project administration (lead); supervision (equal).

## CONFLICT OF INTEREST STATEMENT

All authors have completed the ICMJE uniform disclosure form. There are no conflicts of interest to declare among the authors.

## Supporting information


Tables S1–S5

## Data Availability

The literatures and/or datasets used and/or analyzed during the current study are available from the corresponding author on reasonable request.
